# Characterization of the Body-to-Body Propagation Channel for Subjects during Sports Activities

**DOI:** 10.3390/s18020620

**Published:** 2018-02-18

**Authors:** Marshed Mohamed, Michael Cheffena, Arild Moldsvor

**Affiliations:** Norwegian University of Science and Technology (NTNU), N-2815 Gjøvik, Norway; michael.cheffena@ntnu.no (M.C.); arild.moldsvor@ntnu.no (A.M.)

**Keywords:** body area networks, body-to-body communication, personal communication networks, radio propagation, time-varying channels, performance analysis

## Abstract

Body-to-body wireless networks (BBWNs) have great potential to find applications in team sports activities among others. However, successful design of such systems requires great understanding of the communication channel as the movement of the body components causes time-varying shadowing and fading effects. In this study, we present results of the measurement campaign of BBWN during running and cycling activities. Among others, the results indicated the presence of good and bad states with each state following a specific distribution for the considered propagation scenarios. This motivated the development of two-state semi-Markov model, for simulation of the communication channels. The simulation model was validated using the available measurement data in terms of first and second order statistics and have shown good agreement. The first order statistics obtained from the simulation model as well as the measured results were then used to analyze the performance of the BBWNs channels under running and cycling activities in terms of capacity and outage probability. Cycling channels showed better performance than running, having higher channel capacity and lower outage probability, regardless of the speed of the subjects involved in the measurement campaign.

## 1. Introduction

The aspiration to share information in real-time between co-located body area networks has led to the creation of body-to-body wireless networks (BBWNs). In such a network, wireless devices worn by one person will transmit information wirelessly to a device worn by another person. This kind of communication will find applications in a range of areas such as team sports, emergency services, military as well as other social networking experiences [1]. As in on-body and off-body communications, BBWNs are subject to time-varying body movement and shadowing effects at both ends of the link as all transceivers will be attached to the user in one way or the other. To ensure reliable communication in such time-variant channel conditions, robust hardware and correct decision making tools throughout the protocol stack should be carefully engineered. Such engineering can only be achieved with a greater understanding of the communications channel [2].

An empirical study on the signal characteristics of outdoor body-to-body communication channels was conducted in [3] to assess the impact of typical human body movements. The movements taken into consideration are rotation, tilt, walking in line-of-sight (LOS) and non-line-of-sight (NLOS) conditions. A similar study in an indoor environment was conducted in [4] and it highlighted how a specific movement resulted in different effects on the channel dynamic properties. The study in [5] takes it further, by comparing deterministic and semi-deterministic approaches in the simulation of on-body and body-to-body networks, a semi-deterministic approach was found to be the best option. A more specific study was conducted in [6] to analyze body-to-body communications channels susceptible to shadowed fading. The statistical model proposed in [6] showed an improved fit to the signal fading compared to established models such as Lognormal, Nakagami and Rice. Unlike [6], the work in [2] focused on LOS cases in different indoor environments and showed that the communication channels have considerable variability depending on the local propagation conditions. To mitigate human body shadowing in outdoor body-to-body communication, diversity combining schemes were investigated in [7] and have shown some promising results.

While the studies performed in [2,3,4,5,6,7] cover everyday activities, there has not been significant research into more specific applications such as sport activities. In this paper, for the first time, the body-to-body communication channel characteristics, particularly during running and cycling in an outdoor environment, are investigated. Naturally, these kinds of activities provide their own unique set of channel dynamics due to the movements and posture changes at both ends of the link. This paper presents statistical channel characteristics of two different running and cycling scenarios at the 2.4 GHz industrial, scientific, and medical (ISM) band that is utilized in several standards, which are suitable for short range BBWNs. Due to the imperfection of utilizing single standard distributions in modeling BBWNs channels, [4,8], we investigate the application of the two-state semi-Markov model (TSSMM) in the characterization of such channels. A simulation model based on TSSMM is developed and validated using the measurement data. The measurement results could also provide physical layer parameters for network simulators such as those developed in [5]. Furthermore, we analyze the performance of the investigated scenarios in terms of channel capacity and outage probability and present the results.

The rest of the paper is organized as follows: Section 2 describes the measurement campaign, presenting the body worn transceivers used and the investigated scenarios. Experimental data analysis are discussed in Section 3, followed by simulation model and its validation in Section 4. Performance analysis is presented in Section 5, and Section 6 concludes the paper.

## 2. Measurement Campaign

**Figure 1:**
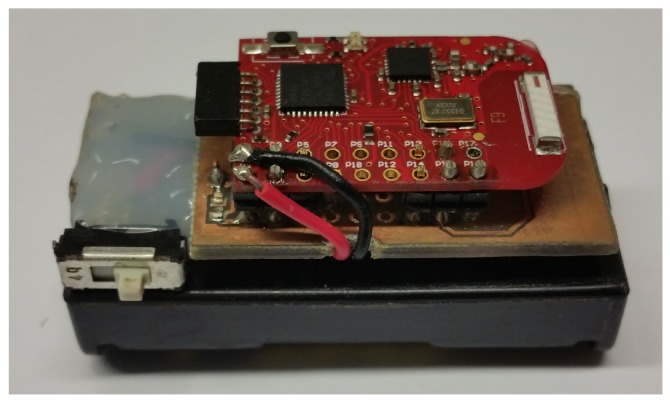
Wearable radio transceiver at 2.4 GHz ISM band. The device is approximately 50 mm × 20 mm × 20 mm.

The BBWN was implemented using one transmitting and one receiving node attached to the upper arm of two adult males of height 1.80 m and mass 80 kg (Subject A) and 1.85 m and mass 75 kg (Subject B), respectively, using a small strip of Velcro. The body location was chosen as it is commonly used for monitoring devices such as mobile phones during sport activities. The experiments conducted in this study were performed in a 500 m’ outdoor stretch, which is part of a common running and cycling route in Gjøvik, Norway. The stretch was bounded by a road on one side and well-spaced industrial buildings on the other. The testbed was a programmable radio transceiver with non-volatile data storage, small enough to be attached on the test subjects (see Figure 1).

**Figure 2:**
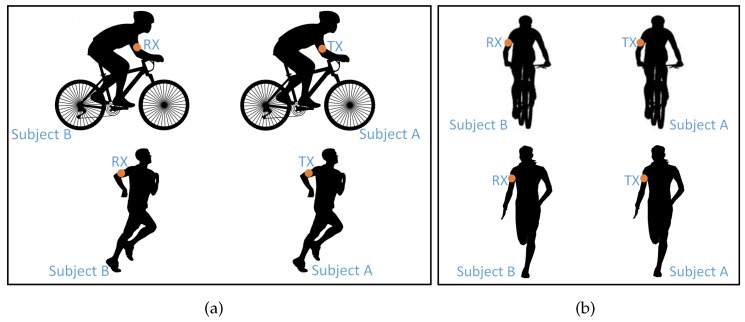
Investigated scenarios of the BBWN measurement campaign. Activities involved were running and cycling. (**a**) Scenario 1, subject behind the other; (**b**) Scenario 2, subject beside the other. The nodes are attached to the side of the upper arms with a radiation pattern away from the body.

It is comprised of a radio transceiver, antenna, microcontroller, microSD memory card and battery. The radio transceiver is CC2500 from Texas Instruments [9]. The nodes use Wurth Electronik Group’s omni-directional chip antenna with a low profile, which is a typical example of antennas to be used in body-mounted transceivers. The device was set to transmit a packet every 4 ms with a constant transmission power of 1 dBm and carrier frequency of 2.425 GHz. This sampling period is way below the estimated coherence time (e.g., 31 ms was reported in [10]) of such channels [10]. At the receiving end, the packet number together with its received signal strength indicator (RSSI) was stored on the MicroSD memory card. The measurement was conducted during two different activities, running at an average speed of 3.33 m/s, and cycling at an average speed of 5 m/s for 500 m. With these conditions, at least 25 kilo-samples for each data set were obtained, which is enough samples for statistical analysis. In each activity, the subjects tried to maintain a separation distance of 1 m between each other. Two different scenarios, which could give an overall representation of the channel dynamics existing in these kind of activities, as shown in Figure 2, were considered:

- Scenario 1, subject behind the other, Figure 2a.
- Scenario 2, subject beside the other, Figure 2b.

## 3. Measurement Results and Analysis

A total of four data sets (two scenarios for each, running and cycling) were collected and analyzed separately. The data set were normalized to their corresponding mean values before they were processed. The data accuracy is limited by the sampling of the RSSI every 4 ms with step size of 0.5 dB. In this section, the time and frequency dynamic characteristics of the measurement results are presented using the time series, auto-correlation functions (ACF) and power spectral density (PSD).

### 3.1. The Time Series

**Figure 3:**
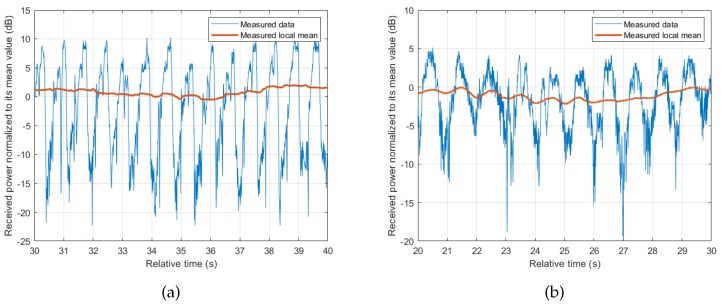
Example of measured time series collected in Scenario 1, (subject behind the other). (**a**) running; (**b**) cycling.

**Figure 4:**
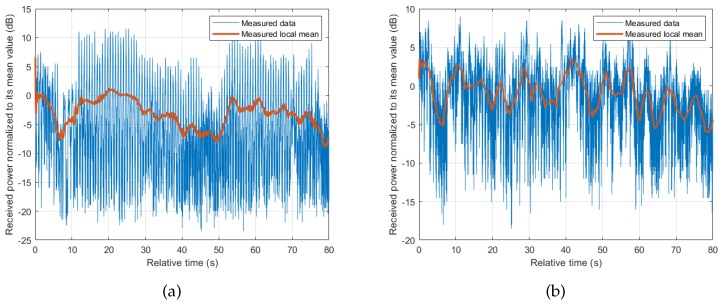
Example of measured time series collected in Scenario 2, (subject beside the other). (**a**) running; (**b**) cycling.

In body-to-body communications, it is hard to categorize the communication link as LOS or NLOS, but more as a periodic transition between the two states. The periodicity depends on the dynamics involved with the specific activity, related to the movements of the body parts and posture changes [11] of the subjects involved. Our collected data has shown that there is a periodic fluctuation of received signal power regardless of the scenario or activity involved. Even when the arms where the transceivers are attached appear to be stationary as in cycling activity, the periodic movement of the body as the subject pedals gives a similar effect. This underlines the significance of the movement of the body in BBWN channels. Figure 3 shows the measured results for Scenario 1, where the periodicity is seen in both running and cycling activities, in which running shows sharper transitions due to movements of the arms. Similar observations are seen in Scenario 2 performed in this measurement campaign. Other contributing factors in the channel properties are the changes in the local environment, and the relative position of the subjects as they perform their activities. This leads to a large scale fading observed by the change in local mean as shown in Figure 4. The local mean was calculated using a sliding window with length of 750 samples, corresponding to 3 s (approximately three cycles of body motion).

### 3.2. The Auto-Correlation Function

**Figure 5:**
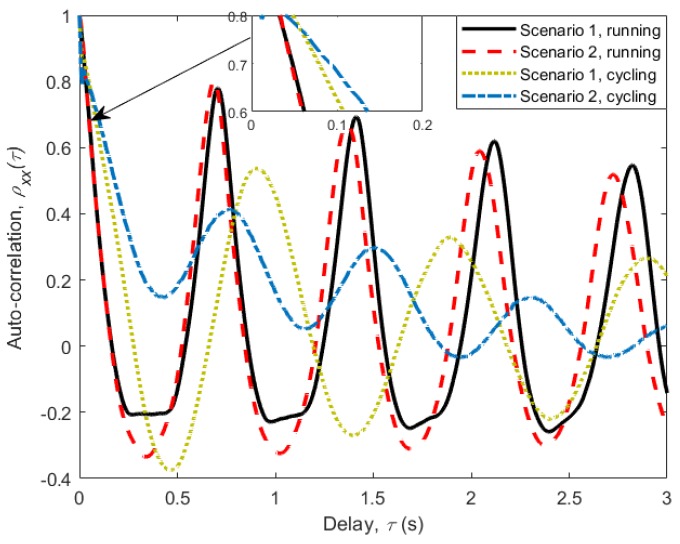
The auto-correlation function of all measured scenarios with a delay of up to 3 s.

One method of characterizing the periodicity in a fading signal envelope is to calculate the auto-correlation function (ACF) of the signal. The ACF provides a useful measure of the degree of time dependency among the observations of stationary signals. For real discrete sampled data x(n), the empirical ACF is given as [12,13]:

(1) $$r_{xx}\left(\tau \right)=\sum_{n=1}^{N-\tau }\left(x\left(n\right)-\mu \right)\left(x\left(n-\tau \right)-\mu \right),$$

where τ is the time delay, *N* is the length and μ is the mean of the sampled data. The correlation was performed over the entire data sample, and was normalized using $r_{xx}\left(0\right)$ to give $\rho_{xx}\left(\tau \right)$. Figure 5 shows plots of the normalized ACF $\rho_{xx}\left(\tau \right)$, for lags of up to 3 s (τ=750 samples). It can be observed that the ACFs resemble exponentially decreasing sinusoids with an approximately constant period. For running activity, the period is consistent with the oscillatory movement of the upper arms where the transceivers are attached, and was found to be approximately 0.7 s. For the case of cycling activity, where there is no backward and forward movement of the upper arm, there still exists a minor periodic movement from leaning and tilting of the body as the subjects pedal the bicycles. This movement is comparatively smaller, and hence can only dominate the ACF in the presence of a permanent LOS condition. In the absence of permanent LOS, such as in Scenario 2 (subject beside the other), other factors such as change in the surrounding environment and subjects’ relative positions start to dominate.

Another important piece of information that can be deduced from the figures depicting ACFs is the channel coherence time $T_{C}$. It is used to describe the time-varying nature of the channel caused by relative motion between the transceivers, and characterizes the frequency selectivity of the channel in time domain. It gives us the lower limit on the transmission rate for the channel not to cause distortion due to motion. It can be defined as the time lag for which the channel correlation coefficient remains above 0.7 [13]. The channel coherence time $T_{C}$ for each scenario and activity are given in Table 1. When the subjects’ motion dominates (e.g., running all scenarios), we observe smaller coherence times. The largest coherent time was 92 ms observed in Scenario 2 (subject beside the other) of cycling.

### 3.3. The Power Spectral Density

**Figure 6:**
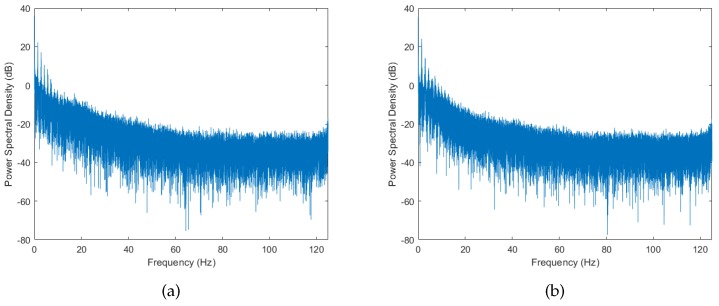
The power spectral density (PSD) of running activity. (**a**) Scenario 1; (**b**) Scenario 2.

**Figure 7:**
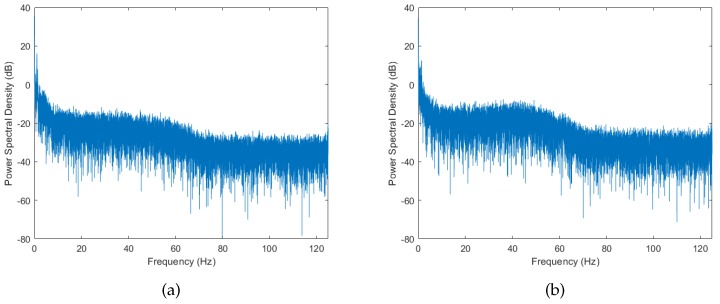
The PSD of cycling activity. (**a**) Scenario 1; (**b**) Scenario 2.

Another method of characterizing dynamic channels is by observing their power spectral density (PSD). The PSD provides useful information on the frequency composition of the signal. It describes how the power of the signal is distributed over the frequency range. Figure 6 and Figure 7 show the PSD for running activity and cycling activities, respectively. We can observe that there is a strong spectral components with high power at 0 Hz followed by other components at higher frequencies in both activities. In addition to that, the PSD tends to decrease exponentially in the logarithmic scale. This lowpass characteristic of the spectrum is consistent with a radio channel having moving scatterers [14], and has been observed in on-body WBAN [15]. As for the case of cycling activity (see Figure 7), the PSD tends to settle between 20 and 50 Hz before it start to decrease again, resulting in a local turning point at around 50 Hz. This phenomenon can be explained by the presence of the off-body scatterer (bicycle) in the cycling activity, which was absent in the running activity. Similar effects of off-body scatterers have been observed for on-body radio channels in [16].

## 4. The Channel Model

It has been observed in Section 3 that the received signal power for BBWNs oscillates between intervals where it is above a certain threshold, and an interval where it is below it (see Figure 3). In addition, the oscillations can be approximated to have a constant period depending on the activity involved. These kinds of channels can be represented using the two-state semi-Markov model (TSSMM) as done in land mobile satellite links [17,18]. In this section, a TSSMM for BBWN channels under various running and cycling scenarios is developed.

We start the modeling process by obtaining the appropriate type of amplitude distribution for the ‘good’ state $D_{g}$, representing an interval in which the received signal is above the local mean, and for the ’bad’ states $D_{b}$, representing an interval in which the received signal is below the local mean. For simplicity, the choice was restricted to the use of same type of distribution for both states. The distribution was determined by obtaining the maximum likelihood (ML) estimates of the measured received signal amplitudes of the two states for Gamma, Lognormal, Nakagami-m, Rayleigh, Rician, and Weibull distributions, and comparing their total negative-log-likelihood. The distribution which gave the largest value of the total negative-log-likelihood was chosen as the best representation of the fading distributions of the two states [19]. It should be noted though that only Scenario 1 (subject behind the other) of cycling activity experience regular line of sight and hence Nakagami-m given by

(2) $$f\left(x|m,\omega \right)=\frac{2m^{m}}{\omega^{m}\Gamma \left(m\right)}x^{2m-1}e^{-\frac{mx^{2}}{\omega }},$$

gave the best representation of the fading distributions for the two states in that scenario. As the LOS path was subjected to shadowing in the other scenarios, Lognormal distribution given by

(3) $$f\left(x|\mu ,\sigma \right)=\frac{1}{x\sigma \sqrt{2\pi }}e^{-\frac{{\left(\ln x-\mu \right)}^{2}}{2\sigma^{2}}},$$

gave the best representation of the fading distributions for the two states instead.

To obtain the time period of each state, we use the obtained ACF shown in Figure 5. From the ACF, one can determine the total period of the two states, which is equal to the oscillation period of the ACF. The ratio of the time period of each state can then be obtained by using the ratio between the samples present in each state. The resulting state periods obtained, $T_{g}$ and $T_{b}$, control the time spent in each state as the state machine alternates between the two states. The simulation model also included the shadowing and fading effects caused by the change in the surrounding environment as well as the changes in the relative position between the subjects (see Figure 4). Since these changes are relatively slow, they are normally considered as large scale fading effects and are modelled using Lognormal distribution [20,21]. The parameters of the distribution were determined by performing ML from the samples of the local mean.

**Figure 8:**
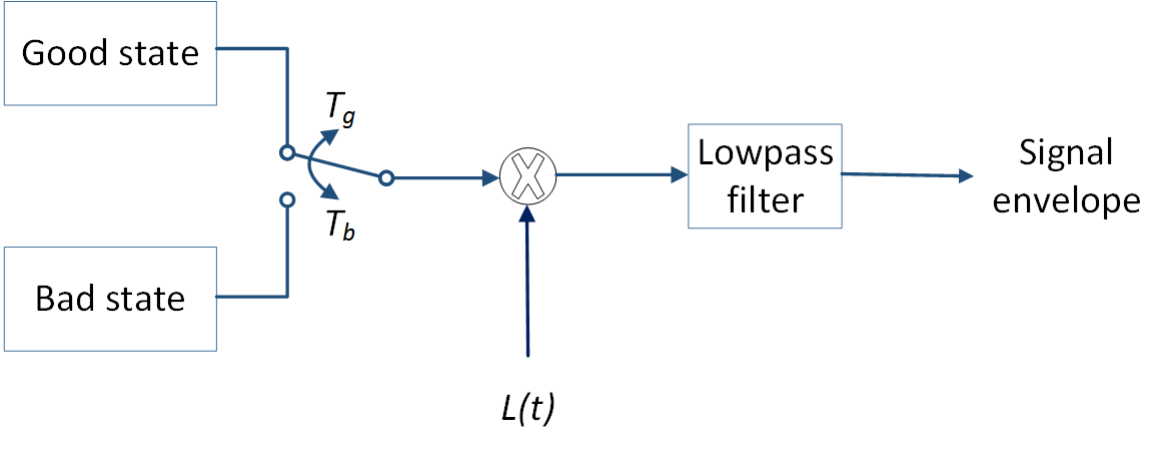
Channel simulator for BBWNs during running and cycling. L(t) is the component of the large scale fading, $T_{g}$ and $T_{b}$ are the period of good and bad states, respectively.

Lastly, to ensure the presence of the right frequency composition, the simulated envelope has to pass through an appropriate filter. The PSD of the measured data shown in Figure 6 and Figure 7 can in general be modeled using a lowpass filter. For the case of running activity, where the PSD decreases exponentially in logarithmic scale (see Figure 6), a first-order Butterworth filter with cutoff frequency around 10 Hz gives a good approximation. For the case of cycling activity, the PSD tend to settle between 20 and 50 Hz before it start decreasing again (see Figure 7). This motivated the use of a third-order Butterworth filter instead, with cutoff frequency around the turning point (50 Hz). Figure 8 summarizes the simulation model for BBWN channels during running and cycling, and the corresponding parameters used in the simulation are given in Table 2 and Table 3. In the simulation model, samples of good and bad states are generated from the corresponding $D_{g}$ and $D_{b}$ distributions for the specified $T_{g}$ and $T_{b}$ periods of time respectively. The outputs are then multiplied by L(t), which is the large-scale fading component. The time series is then passed through the lowpass filter with appropriate cutoff frequency for spectrum shaping. The output from the simulator is a signal envelope that incorporates the overall fading characteristics of body-to-body channels under sporting activities.

**Figure 9:**
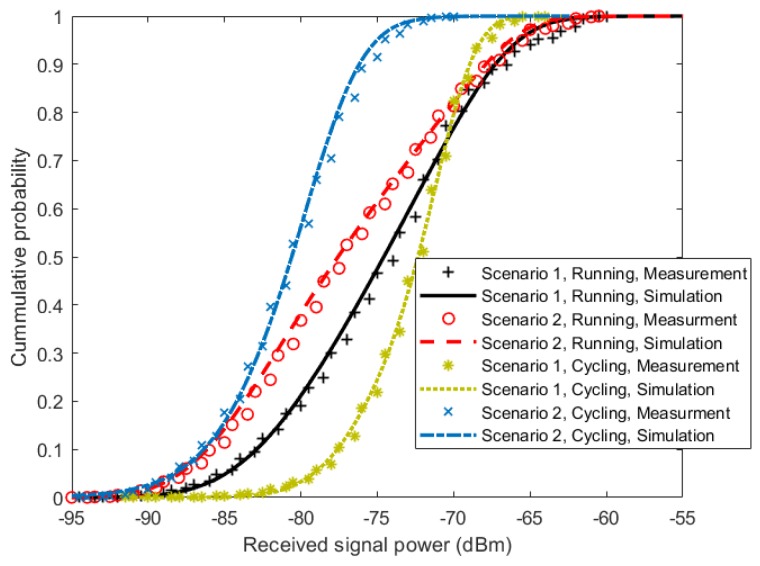
Comparison of cumulative distribution function (CDF) of the measurement results, and the developed simulated model results.

**Figure 10:**
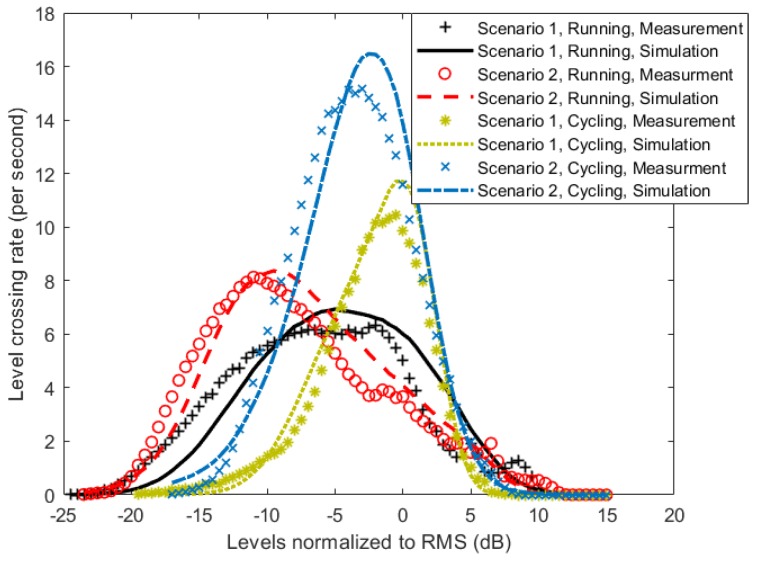
Comparison of level crossing rate (LCR) of the measurement results, and the developed simulated model results.

**Figure 11:**
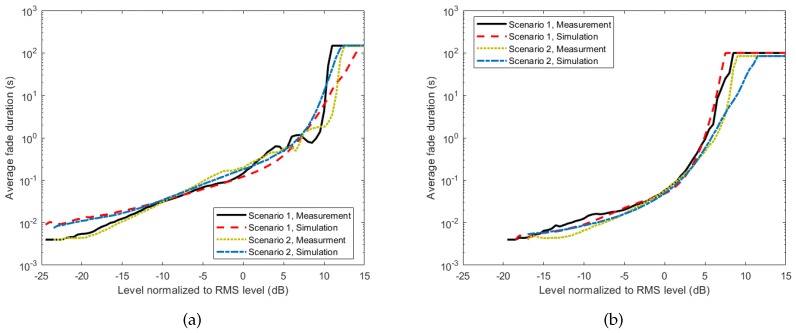
Comparison of AFD of the measurement results, and the developed simulated model results.(**a**) running; (**b**) cycling.

To validate the proposed model, TSSMM channel simulations were performed using parameters given in Table 2 and Table 3, and the first and second order statistics of the received signal power were calculated from the simulation results. The resulting statistics were then compared with the statistics obtained from the measurement data. We start the comparison with the cumulative distribution function (CDF), which is the probability of a signal being below a certain value. This information is commonly used in performance evaluation of the channel with regard to the channel capacity and outage probability. Figure 9 shows a good agreement between the measurement results, and the proposed simulation model results for all scenarios and activities. For second order statistics, the level crossing rate (LCR) and the average fade duration (AFD) were used for comparison. These statistics are important in comparing the time-varying properties of a channel, as they quantify how often the signal crosses a certain threshold and how long it stays below it. Figure 10 and Figure 11 show the comparison of the LCR and AFD of the received signal normalized to their root mean square (RMS) values. We observe a good agreement between the measurement results and the developed simulation model results.

## 5. Performance Analysis

In this section, the TSSMM simulation model of Section 4 is utilized to conduct performance analysis of BBWNs. The analysis is achieved by evaluating the network performance measurement parameters for the two scenarios and the two activities, in terms of channel capacity and outage probability.

### 5.1. Channel Capacity

Channel capacity is the maximum rate at which information can be transmitted reliably over a communication channel. Normalized to the bandwidth, it can be expressed in bps/Hz as [22,23]

(4) $$C_{N}=\log_{2}\left(1+SNR\right),$$

where SNR is the received signal to noise ratio that can be expressed as

(5) $$SNR=\frac{P_{r}}{kTBN_{f}}.$$

**Figure 12:**
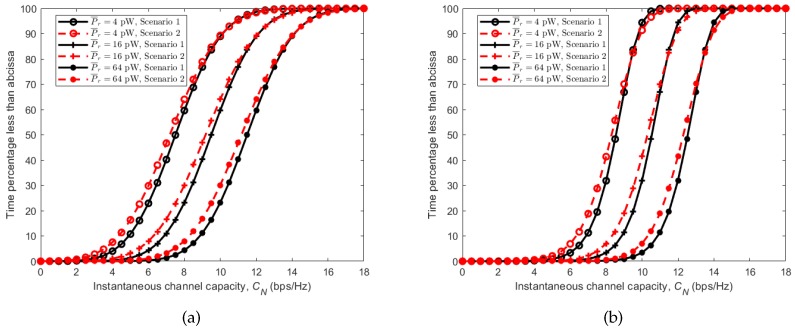
CDFs of channel capacity *C* for different average received power ${\bar{P}}_{r}$. (**a**) running; (**b**) cycling.

Here, $P_{r}$ is the received power, *k* is the Boltzmann constant, *T* is the temperature in Kelvin, *B* is the signal bandwidth in Hz and $N_{f}$ is the receiver linear noise figure. Due to the time-varying fading effects of the received signal power $P_{r}$ in BBWNs, the SNR in (4) varies accordingly, making the channel capacity a random variable. Using the CDFs obtained in Section 4 from the simulation model as well as the measured results, the capacity of the channel in different scenarios and activities were simulated and analyzed. Parameters used to calculate the noise power are given in Table 4. Figure 12 shows the CDFs of the instantaneous channel capacity for different average received power ${\bar{P}}_{r}$. Considering the running activity in Figure 12a, it can be observed that Scenario 1 (subject behind the other) gives the best instantaneous channel capacity most of the time. However, it is interesting to notice that, with equal average received power, the instantaneous channel capacity of the given two possible scenarios is almost equal for more than 25% of the time. This is indicated by the convergence of the two curves in their upper part. With relatively stable transceivers achievable during cycling activity, the instantaneous channel capacities obtained has steeper slopes as seen in Figure 12b. Here, Scenario 1 gives the best instantaneous channel capacity most of the time due to the presence of the LOS component, while Scenario 2 (subject beside the other) gives the worst due to its absence.

**Figure 13:**
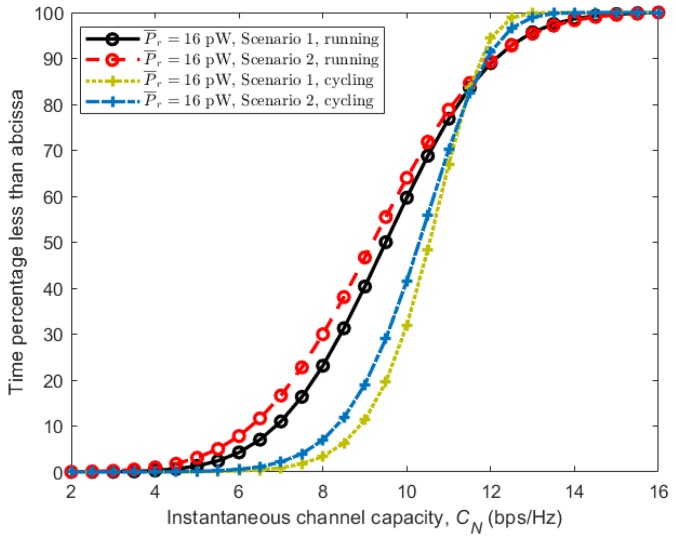
CDFs of channel capacity *C* for all scenarios during running and cycling activities with average received power ${\bar{P}}_{r}=16$ pW.

The effect of the stability of the arms and hence the transceivers can be observed further in Figure 13, where CDFs of instantaneous channel capacity for all scenarios are plotted together. It can be observed that, with similar average received power, cycling where the arm is more stable, outperform running by having larger values in similar scenarios. This is regardless of the fact that the velocity of the subjects during cycling is more than 50% larger than that of running.

### 5.2. Outage Probability

The outage probability $p_{out}$ is defined as the probability that a receiver will not be able to decode the transmitted information correctly. This happens when the received signal has a SNR below a threshold value ${SNR}_{0}$, required for correct decoding, and can be expressed as [23]

(6) $$p_{out}=\mathrm{Pr}\left(SNR\leq SNR_{0}\right).$$

The threshold value ${SNR}_{0}$, depends on the sensitivity of the receiver $S_{r}$, and can be expressed as

(7) $${SNR}_{0}=\frac{S_{r}}{kTBN_{f}}.$$

As in channel capacity, the SNR in (6) varies with the received signal power $P_{r}$, making the outage probability $p_{out}$, a random variable. Again, we use the CDFs obtained in Section 4 from the simulation model as well as the measured results, and values in Table 4 to simulate and analyze the outage probability, relative to the receiver sensitivity $S_{r}$.

**Figure 14:**
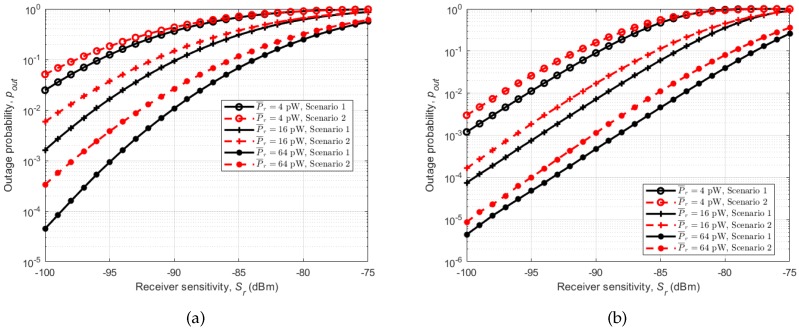
Outage probabilities $p_{out}$ relative to receiver sensitivity $S_{r}$ for different average received power ${\bar{P}}_{r}$. (**a**) running; (**b**) cycling.

**Figure 15:**
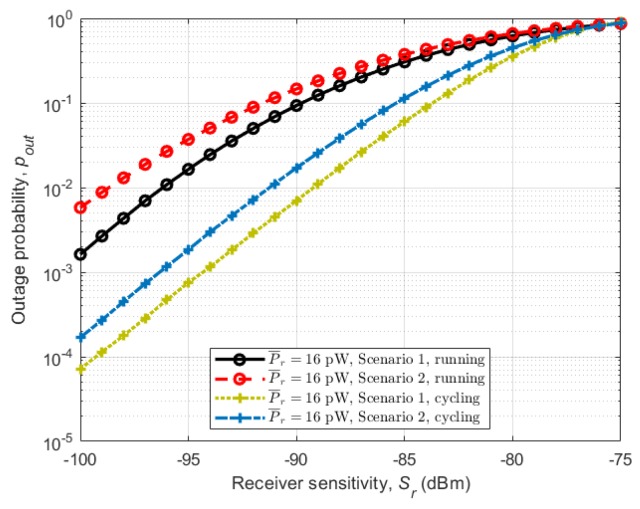
Outage probabilities $p_{out}$ in relation to the receiver sensitivity $S_{r}$ for all scenarios during running and cycling with average received power ${\bar{P}}_{r}=16$ pW.

Figure 14 shows outage probabilities for different average received powers (${\bar{P}}_{r}$) in relation to the receiver sensitivity. Considering the running activity in Figure 14a, we can observe the same trend as in instantaneous channel capacity with Scenario 1 (subject behind the other) having lower outage probability than Scenario 2 (subject beside the other), with the same average received power and receiver sensitivity. This is the same for the cycling activity in which Scenario 1 (subject behind the other) has the lowest outage probability under the same conditions, Figure 14b. As with channel capacity, the effect of the stability of the arms on the performance of the channels is seen in Figure 15, where the outage probabilities of the two activities are plotted together. Cycling shows lower outage probabilities for all scenarios under the same receiver sensitivity.

## 6. Conclusions

This paper presents measurement results of body-to-body communication channels during sport activities. More specifically, the activities involved were running and cycling in an outdoor environment, in which two possible scenarios, which could represent the overall dynamics involved in these kinds of activities, were considered. Due to the on-off nature of the channels in hand, a TSSMM approach was used to develop a simulation model that was validated using the measured data in terms of first and second order statistics, showing good agreement.

The CDFs obtained from the simulation model, as well as the measured results, were then used to conduct performance analysis of the channels in terms of channel capacity and outage probability. Cycling activities showed better performance than running activities for having higher channel capacity and lower outage probability regardless of the speed of cycling being significantly higher. This is mainly because, during cycling, the arms where the transceivers were attached are more stable. The positive effect of the stability of the arms was also seen in LCR in which cycling showed a smaller fading variation compared to running.

In general, these kinds of measurement campaigns and the analysis performed in this study are important in characterizing the transmission channel in hand. They give an aspect to the engineers on the challenges involved in communicating through such channels, and assist in deciding the appropriate solution. Furthermore, the performance analysis completed gives insight, which is important for system design.

## Figures and Tables

**Table 1:** Coherence time: Time before auto-correlation crosses the value of 0.7.

	Coherence Time (ms)	
Scenario	Running	Cycling
Scenario 1	48	80
Scenario 2	48	92

**Table 2:** Parameters used for simulation of running activity.

Parameters	Scenario 1	Scenario 2
Good	Lognormal	Lognormal
distribution, $D_{g}$	μ=0.54,σ=0.61	μ=1.01,σ=0.16
Bad	Lognormal	Lognormal
distribution, $D_{b}$	μ=−1.72,σ=1.32	μ=−1.42,σ=0.93
Large-scale	Lognormal	Lognormal
distribution, *L*	μ=−0.23,σ=0.26	μ=−0.29,σ=0.26
Good period, $T_{g}$	312 ms	256 ms
Bad period, $T_{b}$	392 ms	432 ms
Filter order	First	First
Cutoff frequency, $f_{c}$	8 Hz	15 Hz

**Table 3:** Parameters used for simulation of cycling activity.

Parameters	Scenario 1	Scenario 2
Good	Nakagami-m	Lognormal
distribution, $D_{g}$	m=5.5,ω=1.99	μ=0.29,σ=0.04
Bad	Nakagami-m	Lognormal
distribution, $D_{b}$	m=1.79,ω=0.23	μ=−0.59,σ=0.73
Large-scale	Lognormal	Lognormal
distribution, *L*	μ=−0.05,σ=0.15	μ=−0.14,σ=0.3
Good period, $T_{g}$	436 ms	376 ms
Bad period, $T_{b}$	460 ms	392 ms
Filter order	Third	Third
Cutoff frequency, $f_{c}$	45 Hz	50 Hz

**Table 4:** Parameters used in performance analysis simulations.

Parameter	Value
Average received power (${\bar{P}}_{r}$)	[4 16 64] pW
Boltzmann constant (*k*)	1.38 × 10^−23^ JK^−1^
Temperature in Kelvin (*T*)	290 K
Noise bandwidth (*B*)	300 kHz
Receiver noise figure ($N_{f}$)	6.3
